# Oral flora meningoencephalitis diagnosis by next-generation DNA sequencing

**DOI:** 10.1099/acmi.0.000056

**Published:** 2019-08-19

**Authors:** Eric Heintz, Matthew A. Pettengill, Mohamed Azhar Gangat, Dwight J. Hardy, William Bonnez, Mohammed Mahdee Sobhanie

**Affiliations:** ^1^​ University of Rochester Medical Center, Rochester, NY, USA; ^2^​ Thomas Jefferson University, Philadelphia, PA, USA

**Keywords:** meningoencephalitis, brain abscess, meningitis, oral flora, sequencing

## Abstract

**Introduction.:**

Standard culture methods may fail to detect the causative agents of bacterial infection for various reasons including specimen collection after antibiotic administration, or when standard techniques or environmental conditions are not appropriate for growth of the microorganisms. Conventional 16S rRNA gene sequencing is sometimes a useful alternative technique for identification of bacteria, but is confounded by polymicrobial infection. We present a case of a patient who developed a serious neurological infection for which causative oral flora organisms were observed by microscopy, failed to culture but were identified by next-generation DNA sequencing.

**Case presentation.:**

A male in his forties developed sinus pain and congestion, followed by facial and eye pain, and several weeks later acute-onset confusion and neck stiffness. Cerebrospinal fluid examination revealed pleocytosis and several bacterial morphologies, which were subsequently identified by next-generation sequencing as oral flora constituents *Porphyromonas endodontalis *, *Fusobacterium nucleatum *, *Streptococcus constellatus *, *Prevotella* species and *Parvimonas micra *.

**Conclusion.:**

Oral flora can cause meningoencephalitis and brain abscess formation if translocation occurs by injury or surgical procedures. Next-generation sequencing is often not available at healthcare facilities, or when available may not have been validated for a wide spectrum of specimen sources, but is available at reference laboratories and should be considered when routine methods fail to provide a diagnosis for serious infections.

## Introduction

Next-generation DNA sequencing may provide an aetiological diagnosis in cases where factors including prior antibiotic use, a requirement for non-standard growth conditions or polymicrobial infection complicate routine diagnostic methods. We present below the case of a patient who developed meningitis and encephalitis caused by members of the normal oral flora, which were identified by next-generation DNA sequencing.

## Case Report

A male in his forties developed right frontal sinus pain and congestion, followed several days later by facial and eye pain – more pronounced on the right side. He visited a local emergency department, where a head computed tomography (CT) scan was unremarkable. He was diagnosed with possible migraine, for which he self-medicated with ibuprofen and acetaminophen. His symptoms persisted, and after about 1 week, he began to develop diplopia. He presented to an outside hospital where a head magnetic resonance imaging (MRI) scan was unremarkable. An ophthalmologist reportedly noted a VIth cranial nerve palsy, and the patient was discharged. Several days later, he developed a macular, erythematous, non-vesicular rash on his neck, for which his primary care physician prescribed a 1-week course of valacyclovir that led to resolution of the rash. After about 4 weeks from the onset of his facial symptoms, he presented again to the local emergency department with relatively acute-onset confusion, neck stiffness, urinary incontinence and diaphoresis. A head CT scan showed hydrocephalus. Blood cultures were obtained, but a lumbar puncture was not done before the patient was given one dose of corticosteroids, vancomycin and ceftriaxone, and he was transferred to the emergency department at our institution.

**Fig. 1.**
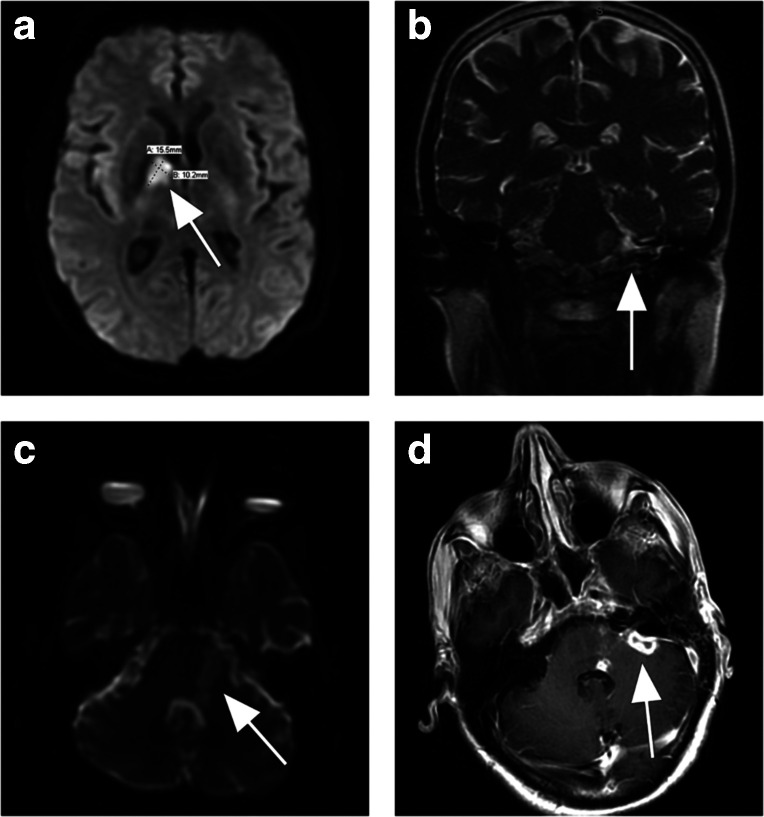
(a) Right anterior thalamic infarct (arrow). (b) Left pontine and cerebellar infarcts (arrow), with leptomeningeal enhancement. (c) Possible microabscesses in left cerebellopontine angle (CPA; arrow). (d) New 13×7 mm consolidative abscess in left CPA cistern (arrow).

Upon presentation, the patient was febrile (39.0 °C) and tachycardic (110 beats per minute). He was obtunded and had a leukocytosis [white blood cell count (WBC) 27.9×10^3^ µl^−^^1^, with 25.5×10^3^ neutrophils µl^−^^1^]. He required intubation for airway protection. A head CT scan did not show any mass or abscesses, but revealed focal decreased attenuation in the basal ganglia. A head MRI and magnetic resonance venography showed patent dural sinuses without evidence of venous thrombosis. There was an area of restricted diffusion in the anterior right thalamus consistent with an acute infarct (Fig. 1a), as well as evidence of multiple scattered foci of probable infarcts in the posterior circulation. In addition, there was a focal area of leptomeningeal enhancement seen along the pons and superolateral left cerebellum consistent with vasculitis associated with meningitis.

Two sets of blood culture bottles showed ‘no growth’ after 5 days of incubation on a BacT/Alert system. Blood cultures collected at the outside hospital were also negative. Cerebrospinal fluid (CSF) was collected by lumbar puncture. Testing showed pleocytosis, elevated protein and decreased glucose concentrations [see Table 1, Day of Care (DOC) 1]. The patient was initially given intravenous acyclovir, vancomycin and cefepime. He was continued on corticosteroids. The CSF PCR was negative for herpes simplex 1 and 2, and varicella-zoster viruses; hence, acyclovir was discontinued. Cytospin Gram stain of the CSF specimen revealed Gram-positive cocci, Gram-negative bacilli and Gram-positive bacilli. Metronidazole was added to the antibiotic regimen to provide coverage for anaerobic organisms. Cryptococcus antigen testing of both CSF and serum was negative. Acid-fast bacilli and fungal stains and cultures were also negative. Lyme disease and human immunodeficiency virus serologies were non-reactive. The aerobic culture of the CSF grew two colonies of α-haemolytic streptococci; however, one of the colonies was not on a streak line and was interpreted as a probable contaminant. Aerobic and anaerobic cultures were repeated on the initial CSF, but failed to grow.

**Table 1.** CSF analysis of specimens collected by lumbar tap on Day of Care (DOC) 1 and 6 Glucose units mg dl^−^^1^, normal range 50–80; protein units mg dl^−^^1^, normal range 15–45 (normal ranges based on patient sex/age). Haematology cell analysis is shown from tube 4 of collection, and similar results were observed from tube 1.

	Glucose	Protein	WBC	RBC	%Neut	%Lymph	%Mono	%Mac
DOC-1	16	279	6080	1357	69	25	6	0
DOC-6	72	144	171	119	7	91	0	2

**Fig. 2.**
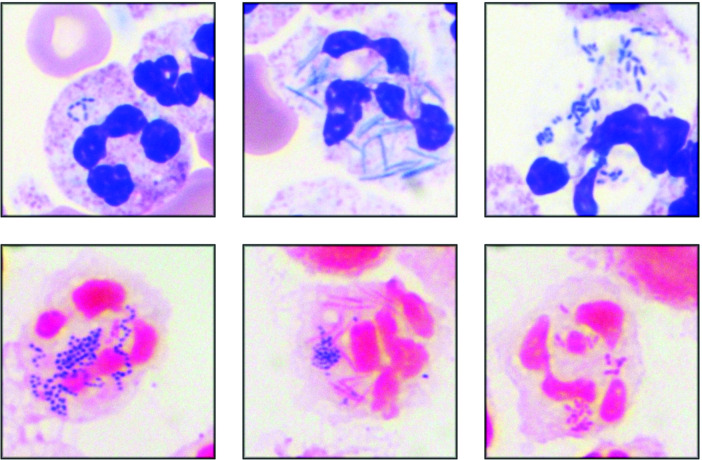
Wright-Giemsa (upper panels) and Gram stain (lower panels) microscopy of CSF collected on DOC 1.

Because bacterial organisms were seen by Wright-Giemsa stains and Gram stains of the CSF (see Fig. 2), but no organisms grew in culture, we submitted the specimen to the University of Washington Molecular Microbiology Laboratory for universal bacterial PCR/sequencing. The patient CSF specimen generated a mixed signal from Sanger sequencing, after which next-generation sequencing was performed, which revealed a major quantity of *Porphyromonas endodontalis *, moderate quantity of *Fusobacterium nucleatum *, and minor quantity of *Streptococcus constellatus *, *Prevotella* species (related to *Prevotella pleuritidis *) and *Parvimonas micra *. When the results of this testing became available, the vancomycin treatment was discontinued and the patient was maintained on cefepime and metronidazole.

The patient’s mental status improved, allowing for extubation on hospital day 3. He then complained of persistent occipital headache, neck pain and poor hearing in the left ear. His neurological exam was notable for left gaze paresis consistent with VIth nerve palsy. In addition, he had left VIIth palsy with left-sided weakness and numbness. He was noted to have dysmetria with finger-to-nose testing of the left upper extremity, but heel-to-shin coordination was intact bilaterally. A head MRI scan on day 8 showed progression of prior thalamic and posterior infarcts, a new punctate infarct in the left ventral medulla, stable leptomeningeal disease (Fig. 1b) and stable areas of restricted diffusion along the left cerebropontine angle, consistent with microabscesses (Fig. 1c). Transoesophageal echocardiography showed no evidence of valvular vegetations or right-to-left shunting.

CSF collected again on DOC 6 – before the sequencing results were reported – showed continued pleocytosis, although considerably reduced in magnitude from DOC 1 (see Table 1). Other parameters also indicated improvement.

The patient was transferred to an inpatient rehabilitation unit where he continued to receive intravenous antibiotics, as his mobility and diplopia slowly improved. There, he was evaluated by a speech/swallow pathologist who noted pocketing of retained food in his mouth. Oral exam was notable for poor dental hygiene, with several missing lower teeth, and use of dentures in place of upper row teeth. None of the remaining lower teeth appeared to be loose. After completing approximately 3 weeks of antibiotics, he again underwent a head MRI scan, which showed no meningeal abnormality, but new subtle region of infarction in the left cerebellar peduncle, and a new consolidative abscess in the left cerebellopontine angle cistern, measuring 13×7 mm (Fig. 1d). The Neurosurgery Department was consulted for this latter finding, but noted no signs of encephalopathy or meningismus, and felt that the collection was due to an indolent process not necessitating acute surgical intervention. He remained haemodynamically stable. We decided to extend his antibiotic course for another 3 weeks, with repeat imaging to assess the resolution of the abscess. Cefepime was narrowed to ceftriaxone and oral metronidazole was continued. He was discharged to home after approximately 1 month of hospitalization. Repeat head MRI was performed approximately 2 weeks later, showing resolution of the abscess. The antibiotic treatment was stopped.

We saw the patient in the infectious diseases’ clinic about 1 month after the cessation of antibiotics. He reported continued poor hearing in the left ear, but improvement in the left-sided facial weakness. Diplopia had resolved by that point, but he was noted to have some gaze nystagmus in both horizontal directions. He continued to have slight dysmetria of the left upper extremity. His inflammatory markers, WBC and electrolytes remained stable and within normal limits. A follow-up CT angiography done at this visit showed stable chronic infarcts involving the right basal ganglion, thalamus and left lateral pons. There were no signs of development of new peripheral abscess, intra-arterial thrombus or aneurysm. The patient was continued on aspirin for secondary stroke prevention.

## Discussion

Bacterial meningitis remains a significant cause of morbidity and mortality despite the fact that its rates have dropped significantly in regions with successful implementation of vaccination campaigns against *Streptococcus pneumonia* and type B *Haemophilus influenzae* [1, 2]. Meningitis or encephalitis due to seeding of the CNS with common oral [3–7] or colonic [8, 9] floral anaerobic organisms has been described before in case reports, but the application of next-generation sequencing to clinical specimens has made aetiological identifications possible in highly polymicrobic, culture-negative, bacterial meningitis cases, such as presented with our patient. The methods of the specific assay performed by a reference laboratory in our case, and the application of these methods to polymicrobial infections in general, was recently reported [10] and included 16S rRNA deep sequencing (Illumina MiSeq) utilizing the primers and databases published previously [11] (primers available in an open access supplementary file).

Our patient’s CSF was invaded by a spectrum of organisms that commonly inhabit the oral cavity. The genera *Porphyromonas *, *Fusobacterium *, *Streptococcus* and *Prevotella* are normal flora in the healthy oral space [12–14]. Additionally, some of the organisms detected in our patient have been associated with various dental diseases. *Parvimonas micra* [15] and *Porphyromonas endodontalis* [16] have been associated with dental root canal infections. *Fusobacterium nucleatum* has been implicated in several oral diseases [17], including Lemierre syndrome, as well as causing other systemic infections including bacteraemia. It is not possible to discern which of the organisms detected in our patient’s CSF were involved in infarction and abscess development. Our patient was noted to have poor dental hygiene, with several missing teeth. However, no teeth appeared to be loose, and CT imaging did not suggest any dental caries or abscess.

Laboratory testing of CSF does not routinely include anaerobic culture. Therefore, physicians should strongly consider requesting anaerobic culture and altering patient treatment to include anaerobic antimicrobial coverage if polymicrobial infection is observed by microscopy or there is evidence of any injury which may cause communication between the oral space and CNS. When culture methods fail to yield an aetiological answer, and further microbiological evaluation is justified, broad-range bacterial 16S rRNA PCR and sequencing may help to provide a diagnosis. In this case, the highly polymicrobial nature of the infection required the use of next-generation DNA sequencing. We submitted the specimen on the 6th day of hospitalization, and received the sequencing results from the reference laboratory on the 13th day of hospitalization. Despite the considerable time to result reporting, the results led to a change in patient antimicrobial management. When organism identifications cannot be provided by routine laboratory methods for serious infections, advanced technologies including next-generation sequencing should be employed.
